# The Ectopic Expression of *Btr2* in *Aegilops tauschii* Switches the Disarticulation Layer From Above to Below the Rachis Node

**DOI:** 10.3389/fpls.2020.582622

**Published:** 2020-11-09

**Authors:** Xiaoxue Zeng, Akemi Tagiri, Shinji Kikuchi, Hidenori Sassa, Takao Komatsuda

**Affiliations:** ^1^Institute of Crop Science, National Agriculture and Food Research Organization (NARO), Tsukuba, Japan; ^2^Graduate School of Horticulture, Chiba University, Matsudo, Chiba, Japan

**Keywords:** triticeae, barrel type dispersal units, lignin, cell wall, abscission zone, ectopic expression

## Abstract

Seed dispersal among wild species belonging to the tribe Triticeae is typically achieved by the formation of a brittle rachis. The trait relies on the development of a disarticulation layer, most frequently above the rachis node (resulting in wedge type dispersal units), but in some species below the rachis node (resulting in barrel type dispersal units). The genes responsible for the former type are the complementary pair *Btr1* and *Btr2*, while the genetic basis of the latter type has yet to be determined. *Aegilops tauschii* forms barrel type dispersal units and previous study showed this species lacked an intact copy of *Btr1*. Here it has been demonstrated that *Ae. tauschii* carries two of *Btr2*; and that *Btr2* transcript is present in a region below the rachis node where the abscission zone forms. The implication is that in this species, the *Btr2* product is involved in the formation of barrel type dispersal units.

## Introduction

Many wild grass species ensure the dispersal of their seed by their inflorescence becoming brittle at physiological maturity; the trait requires the formation of one or multiple abscission zones (Cousens et al., 2008). The anatomy and positioning of the abscission zone varies from species to species, although not in a way which necessarily correlates with phylogeny (Yu et al., 2020b). Among species belonging to the Triticeae tribe, the zone forms either in the rachis or the rachilla (Sakuma et al., 2011). Rachis disarticulation most frequently occurs above the node, but in a few cases (as in, for example, *Aegilops tauschii*) it can occur below the node (Zohary et al., 2012).

Disarticulation above the node results in the formation of wedge type dispersal units, each consisting of a single spikelet attached to a short proximal section of the rachis. The presence on chromosome arm 3HS of the dominant, complementary and tightly linked genes *Btr1* and *Btr2* is necessary for the expression of the trait in wild forms of *Hordeum vulgare* (barley; Pourkheirandish et al., 2015). In *Triticum* and *Aegilops* species, *Btr1* and *Btr2* map to the short arm of the group 3 homeolog (Avni et al., 2017; Zeng et al., 2020b). The products of this pair of genes are presumed to determine the unique mode of disarticulation exhibited by the *Aegilops longissima* spike, in which only one or two of the central nodes are brittle (Zeng et al., 2020a). An alteration in the *Btr1* coding region sequence is thought to be responsible for the non-brittle nature of the spikes formed by both einkorn (*Triticum monococcum*; Pourkheirandish et al., 2018; Zhao et al., 2019) and emmer (*Triticum dicoccum*; Avni et al., 2017) wheat. The transcription of *Btr2* above the rachis node has been suggested as a critical determinant of the formation of a disarticulation zone in both barley (Pourkheirandish et al., 2015) and *Ae. longissima* (Zeng et al., 2020a).

The barrel type dispersal unit consists of a single spikelet attached to a short distal rachis segment; among *Aegilops* species, this form is only found in species harboring the D genome (van Slageren, 1994). According to Chen (2001), F_1_ hybrids formed by crossing a weedy form of *Triticum aestivum* forming wedge type dispersal units with *Triticum spelta* (barrel type units) disarticulate both above and below the rachis node, suggesting that different gene(s) underlie the formation of the two forms of dispersal unit. The genetic basis of the barrel type unit was found to be a locus mapping to chromosome arm 3DL (Li and Gill, 2006; Zhang et al., 2015). The locus harbored a homolog of rice gene *qSH1*, *TaqSH1-D* (Katkout et al., 2015), which encodes a BELL1-type homeobox protein implicated in the shattering phenotype (Konishi et al., 2006). An insertion present in the *TaqSH1-D* 3'-UTR has been proposed to be the genetic basis of rachis non-brittleness, although this hypothesis awaits verification. As yet, the genetic basis of the barrel type dispersal unit has yet to be identified.

The geographical range of the diploid species *Ae. tauschii*, the donor of the D genome harbored by bread wheat (*T. aestivum*), stretches across western and central Asia into south eastern Europe (van Slageren, 1994). Two phylogenetic lineages (L1 and L2) of the species have been recognized (Wang et al., 2013): the former includes accessions of ssp. *tauschii*, while the latter includes both ssp. *tauschii* and ssp. *strangulata accessions*. As it has been suggested that the L2 type is the one more closely related to the bread wheat D genome (Wang et al., 2013), and L2 representative has been selected for whole genome sequencing (Jia et al., 2013; Luo et al., 2017).

In both rice and sorghum, the products of multiple genes are known to determine seed shattering (Yu and Kellogg, 2018; Di Vittori et al., 2019). A comparison of the *Btr1* and *Btr2* sequences harbored by a range of grass species has shown that while *Ae. tauschii* lacks an intact copy of *Btr1*, two copies of *Btr2* are present, both of which map along chromosome arm 3DS (Zeng et al., 2020b). Since *Btr1* and *Btr2* act as a complementary gene pair in most Triticeae species, *Ae. tauschii* represents an anomaly, raising the question as to whether the products of the two *Btr2* genes underly the formation of the species’ barrel-type disarticulation units. The present experiments sought to establish the involvement, if any, of the *Btr2* product in the formation of barrel-type units by *Ae. tauschii*.

## Materials and Methods

### Plant Materials

Accessions of each of *Ae. tauschii* ssp. *tauschii* (AE 956) and *Ae. longissima* ssp. *longissima* (AE 417) were obtained from the IPK Genebank.^1^ Plants were grown in a glasshouse at the National Institute of Crop Science (Tsukuba, Japan).

### Histological and Anatomical Analysis

Spikelets were sectioned longitudinally by hand and stained by immersion for 3 h in 0.01% w/v acridine orange in distilled water, then rinsed in 10x PBS buffer (pH7; Acridine orange stains lignin green and anionic polysaccharides red, see Li and Reeve (2005) and Houtman et al. (2016). The sections were illuminated with a 488 nm diode laser and the output was captured using a confocal laser scanning microscope (LSM 700, Carl Zeiss, Tokyo, Japan) equipped with ZEN 2009 Light Edition CLSM software, scanning at 505–530 nm (green) and >600 nm (red). The surface of the rachis separation layer in mature dispersal units was characterized using a TM3000 scanning electron microscope (SEM; Hitachi, Tokyo, Japan), set to deliver an accelerating voltage of 1 kV.

### DNA Sequence Analysis

Genomic DNA was extracted from fresh leaves following Komatsuda et al. (1998). The subsequent PCR amplification and DNA sequencing procedures followed those given by Pourkheirandish et al. (2015). CLC Sequences Viewer v8 software was used to align sequences.^2^

### Quantitative RT-PCR

Three replicates, each consisting of three 0.5–1.5 cm long spikes at the white anther stage, were sampled (Kirby and Appleyard, 1981), and RNA was extracted from these using the TRIzol reagent (Invitrogen, Carlsbad, CA, United States). After treatment with RNase-free DNase (Takara Bio, Kusatsu, Japan) and quantification using a NanoDrop 1,000 device (Thermo Scientific, Tokyo, Japan), a 1 μg aliquot was reverse-transcribed using SuperScript III (Invitrogen), and the resulting ss cDNA used as template for quantitative PCRs (qPCRs) based on the Thunderbird SYBR qPCR Mix kit (TOYOBO, Tokyo, Japan) and a CFX96 real-time PCR system (Bio-Rad, Tokyo, Japan). Each sample was represented by a minimum of three technical replicates.

### RNA *in situ* Hybridization

Both an anti-sense and sense version of a segment of the *Btr2* sequences were generated by PCR from *Ae. longissima* accession AE 417 (Zeng et al., 2020a). The PCR amplicons were purified using a QIAquick PCR purification kit (Qiagen, Germantown, MD, United States). One of the probes for *Btr2-Lo-1* (identical to *Btr2-D-1*) covered 150 bp of the *Btr2* coding region and 200 bp of its 3'-UTR, while the other one for *Btr2-Lo-2* (identical to *Btr2-D-2*) covered 150 bp of the *Btr2* coding region and 150 bp of its 3'-UTR. Adaptor sequences including promoter of T7 RNA polymerases (CGCGCGTAATACGACTCACTATAGGG) were added to the 5' end of the primers. The amplicons were validated by DNA sequencing before use. Hybridization probes were prepared using T7 RNA polymerase. The RNA *in situ* hybridization procedure, based on digoxigenin-labeled RNA probes, was performed on samples harvested at the white anther stage, following methods given by Komatsuda et al. (2007).

## Results

### Disarticulation in the Mature Spikes of *Aegilops tauschii* and *Aegilops longissima*

**Figure 1:**
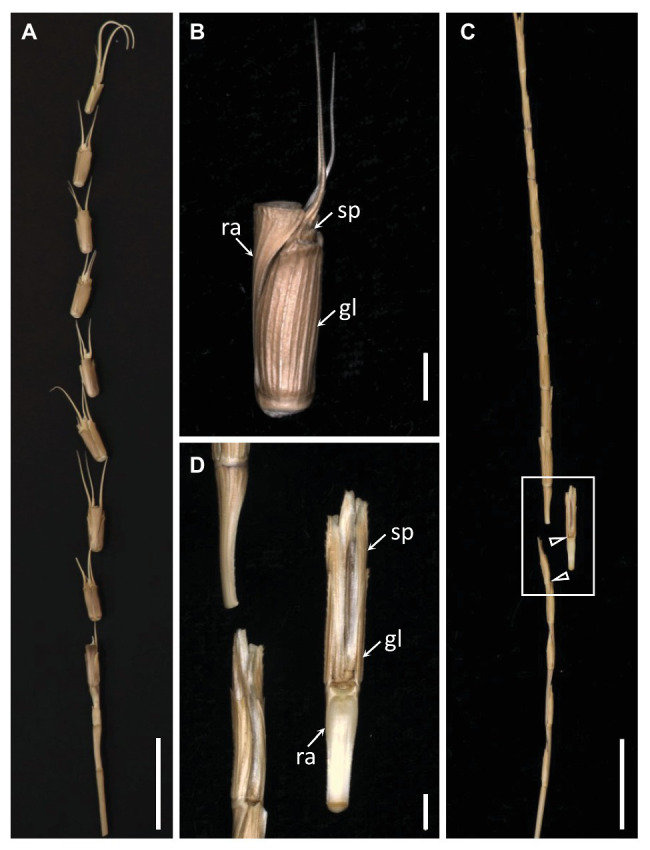
The dispersal units of *Aegilops tauschii* and *Aegilops longissima*. **(A)** The mature *Ae. tauschii* spike, which produces **(B)** barrel type dispersal units comprising one spikelet attached to a distal rachis segment. **(C)** The mature *Ae. longissima* spike produces **(D)** wedge type dispersal units comprising one spikelet attached to a proximal rachis segment. Arrowheads indicate brittle positions. sp, spikelet; gl, glume; ra, rachis. Scale bars: 2 cm in **(A)** and **(C)**, 2 mm in **(B)** and **(D)**.

**Figure 2:**
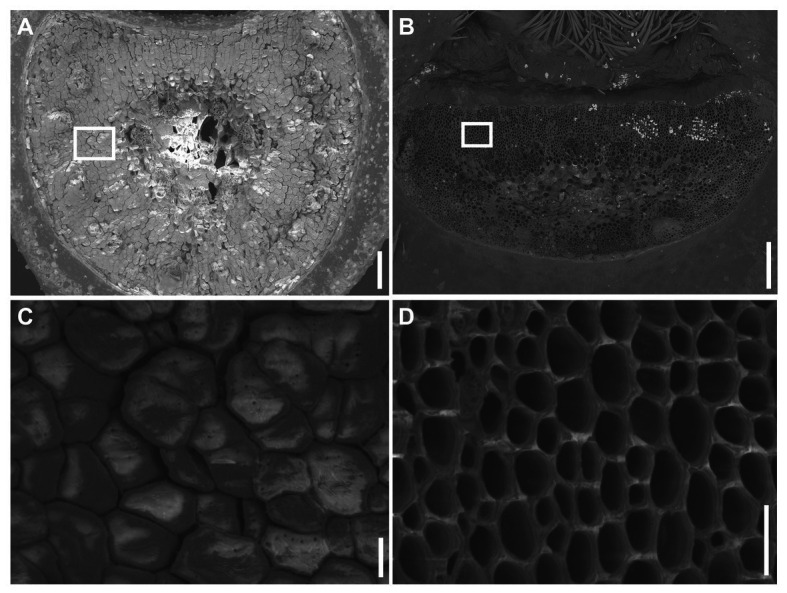
SEM visualization of the disarticulation surface. **(A)** The disarticulation surface below the rachis node in *Ae. tauschii* has a smooth surface, with all but the vascular cells being capped by intact cell wall **(C)**. **(B)** The disarticulation surface above a rachis node in *Ae. longissima* is smooth, but all the cells are uncapped by cell walls **(D)**. Scale bars: 200 μm in **(A)** and **(B)**, 20 μm in **(C)** and **(D)**.

**Figure 3:**
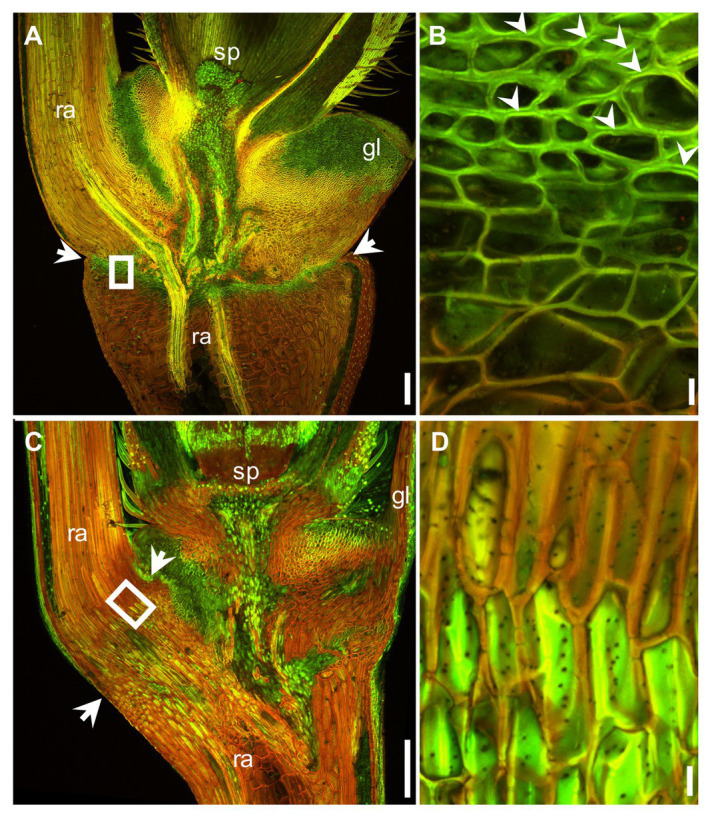
Acridine orange staining of the region around the abscission zone imaged at anthesis using LSM. The output of the green (505–530 nm) and red (>600 nm) channels have been merged. Lignin appears green and anionic polysaccharides red. Longitudinal section across the basal part of a floret and a section of the rachis in *Ae. tauschii* **(A)** and *Ae. longissima* **(C)**. The abscission zone is arrowed. **(B)** and **(D)** A magnified view of the region shown in **(A)** and **(C)** by a white box. The preferential deposition of lignin in the secondary wall of the abscission zone is indicated by the arrowheads shown in **(B)**. sp.: spikelet, gl: glume, ra: rachis. Scale bars: 200 μm in **(A)** and **(C)**, 10 μm in **(B)** and **(D)**.

The mature *Ae. tauschii* spike was brittle (Figure 1A), disarticulating below each rachis node, thereby generating barrel type dispersal units (Figure 1B). In contrast, disarticulation in the *Ae. longissima* spike occurred above two nodes in the central portion of the rachis, resulting in the production of wedge type units (Figures 1C,D). The surface of the separation layer in the *Ae. tauschii* spike was smooth, as a result of all but the non-vascular cells having been capped by an intact cell wall (Figures 2A,C). In *Ae. longissima*, by way of contrast, although the surface of separation layer was also smooth, few cells were capped by a cell wall (Figures 2B,D). A typical LSM image of a stained longitudinal section of *Ae. tauschii* is shown in Figure 3A. No autofluorescence was observed in either the green or the red channel (data not shown), but following staining with acridine orange, there was evidence of lignification in cells lying below the rachis node (Figure 3A). In particular, lignin accumulated in about four layers of small cells within the abscission zone (Supplementary Figure S1A), while the accumulation of anionic polysaccharides in this zone was relatively limited (Supplementary Figure S1C). The lignin was preferentially deposited in the secondary wall (Figure 3B). No such cells were observed in the *Ae. longissima* disarticulation layer (Figures 3C,D), even in a higher magnification (Supplementary Figure S2), implying the absence of a distinct abscission zone.

### *Aegilops tauschii* Harbors Two Copies of *Btr2*

**Figure 4:**
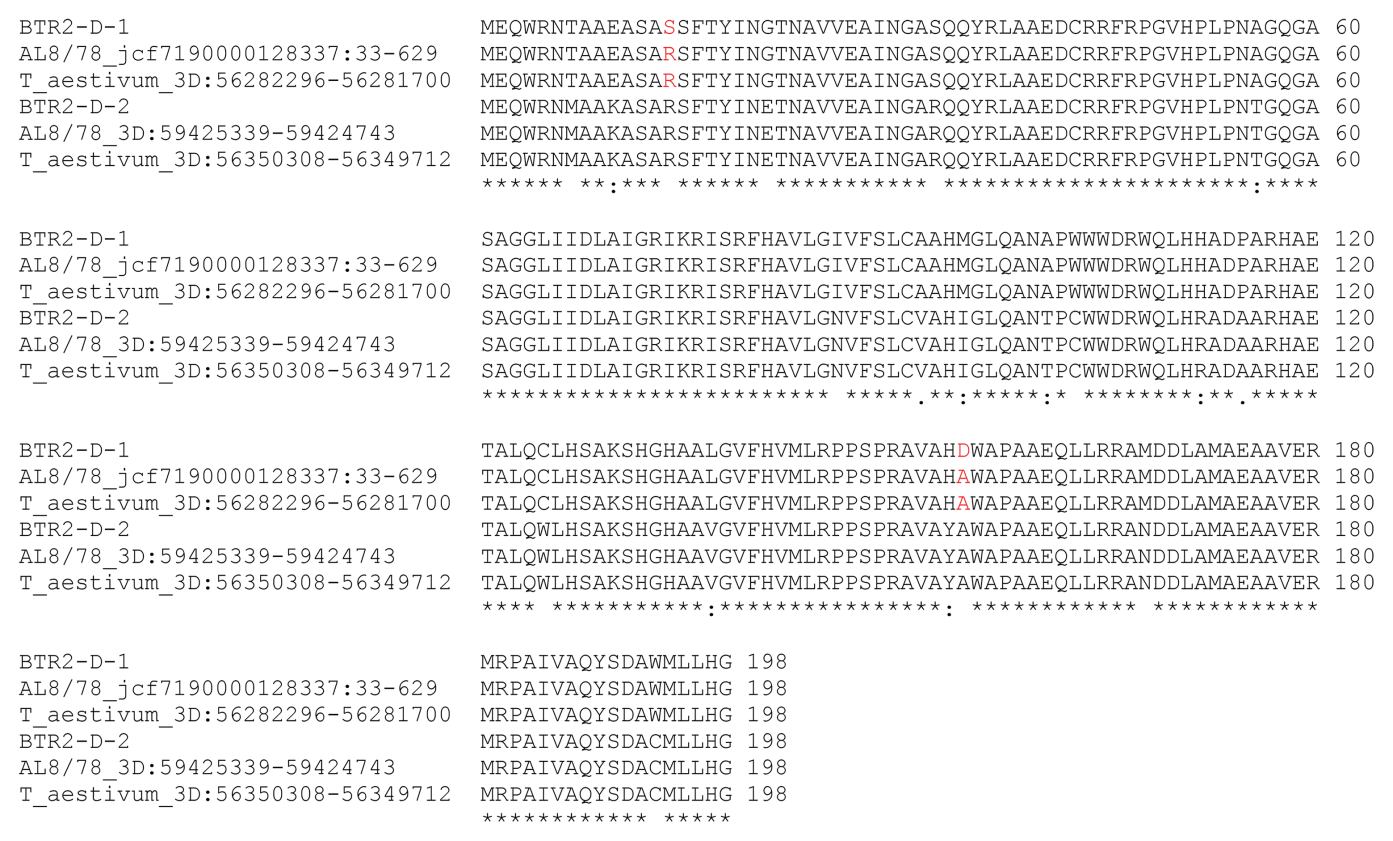
Alignment of the deduced polypeptide sequences encoded by *Btr2*s of the *Ae. tauschii* ssp. *tauschii*, ssp. *strangulata* and a *Triticum aestivum*. Keys below indicate conserved sequence (*), conservative mutations (:), semi-conservative mutations (.), and non-conservative mutations ().

The *Ae. tauschii* accession AE 956 harbored two copies of *Btr2*, namely *Btr2-D-1* (GenBank accession MT920643) and *Btr2-D-2* (GenBank accession MT920644). *Btr2-D-1* of AE 956 corresponds to the *Ae. tauschii* accession AL8/78 sequence jcf7190000128337: 33-629, and *Btr2-D-2* of AE 956 corresponds to the *Ae. tauschii* accession AL8/78 sequence 3D: 59425339-59424743. The level of homology between *Btr2-D-1* of AE 956 and its AL8/78 equivalent was 99.5%, and the homology between *Btr2-D-2* of AE 956 and its AL8/78 equivalent was 100% (Supplementary Figure S3). Both AE 956 genes included a 597 nt coding region (Supplementary Figure S3) encoding a 198 residue protein (Figure 4). The two genes differed from one another at 29 nucleotide positions (Supplementary Figure S3), resulting in 19 polymorphisms at the polypeptide level (Figure 4). The Triticeae *Btr2* sequences most closely related to *Btr2-D-1* and *-D-2* are, respectively, the *Ae. longissima* accession AE 417 homologs *Btr2-Lo-1* (94% homology) and *Btr2-Lo-2* (97% homology; Zeng et al., 2020a). A recent duplication in the genome of the common ancestor of *Ae. longissima* and *Ae. tauschii* ssp. *strangulata* is thought to be responsible for the presence of two copies of *Btr2* (Zeng et al., 2020b). Both *Btr2* copies of *Ae. tauschii* ssp. *strangulata* are almost identical to those in *Ae. tauschii* ssp. *tauschii* except for two amino acid substitutions (Figure 4). The D genome of bread wheat (hexaploid; Alaux et al., 2018) has retained both *Btr2* copies; their sequences in the model cultivar Chinese Spring are identical to those in *Ae. tauschii* ssp. *strangulata* (Figure 4). Inspection of the genome sequence of 10 other bread wheat cultivars available at https://webblast.ipk-gatersleben.de/wheat_ten_genomes/viroblast.php shows the same result. The origin of *Btr2* and *Btr2*-like dates back to an ancient duplication event (Pourkheirandish et al., 2015), which occurred in the common ancestor of the Triticeae tribe (Zeng et al., 2020b).

### The Profile of *Btr2* Transcription in *Aegilops tauschii*

**Figure 5:**
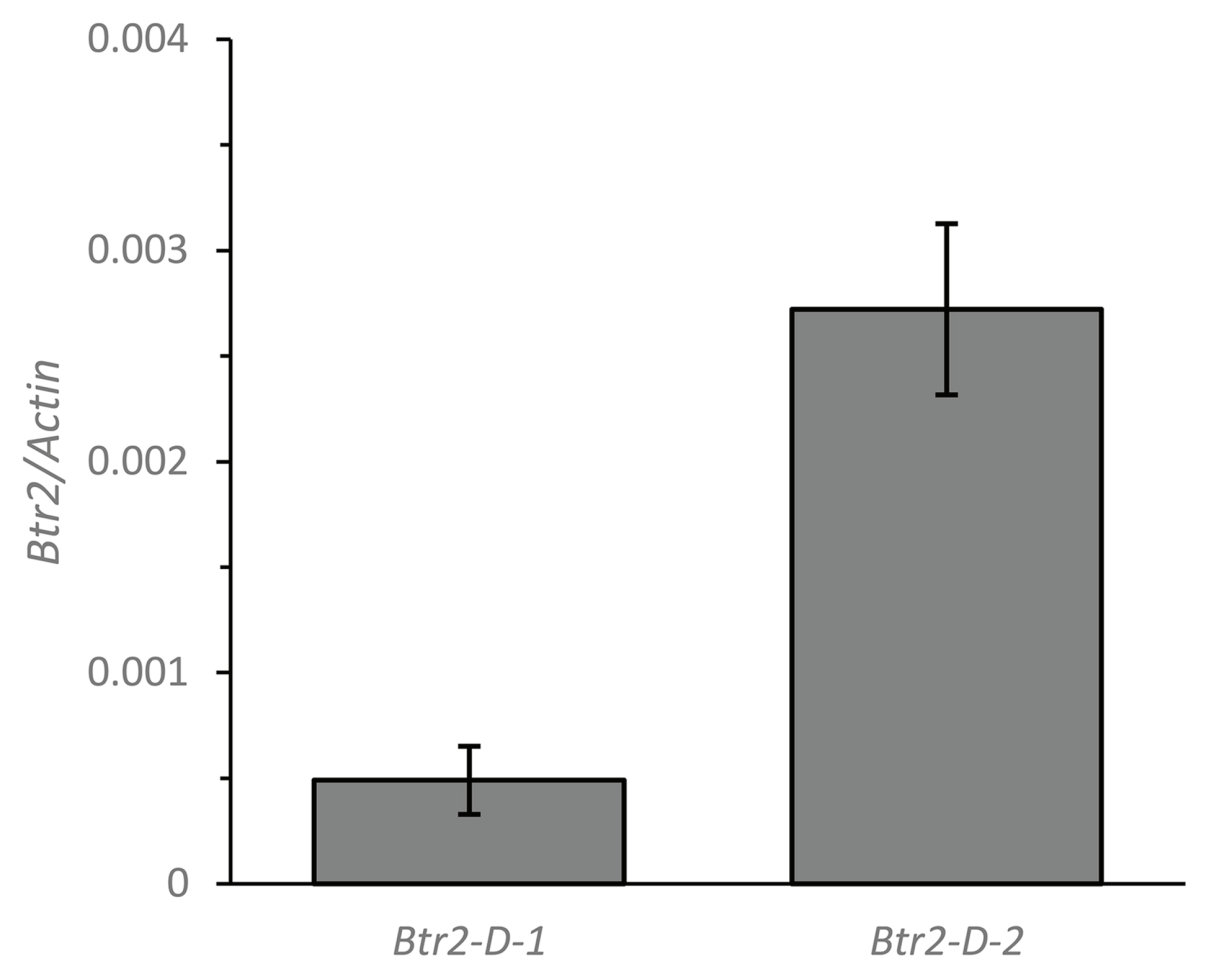
The abundance of *Btr2* transcript, estimated from a quantitative PCR (qPCR) assay, in the spike of *Ae. tauschii* sampled at the white anther stage. Data shown in the form mean ± SE (*n* = 3).

**Figure 6:**
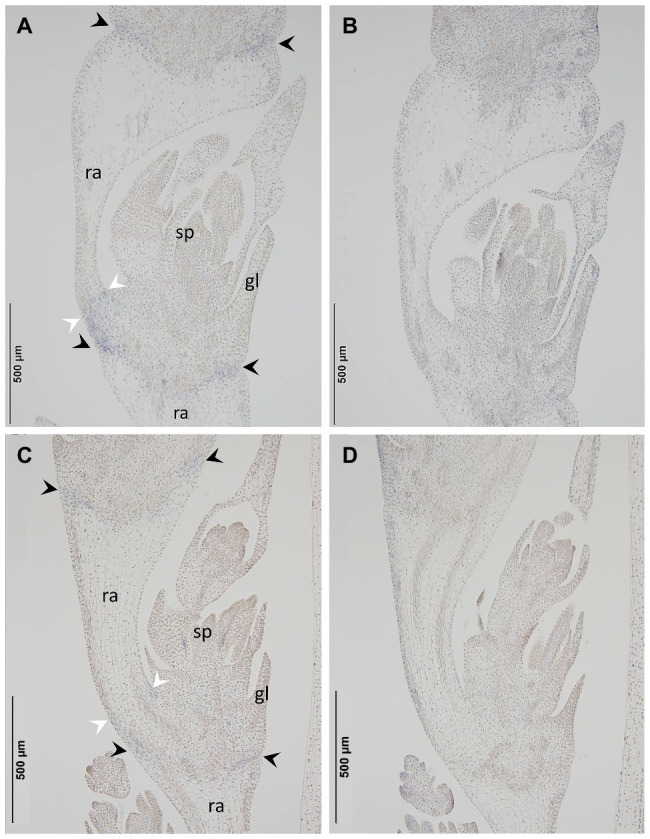
The site in the *Ae. tauschii* spike where *Btr2* transcript accumulates, as assayed using RNA *in situ* hybridization. The probes used were **(A)** *Btr2-D-1* antisense, **(B)** *Btr2-D-1* sense, **(C)** *Btr2-D-2* antisense, **(D)** *Btr2-D-2* sense. Signal was detected between the spikelet and the rachis node (black arrows) and above the rachis node (white arrows) in **(A)** and **(C)**, but not in **(B)** or **(D)**. sp, spikelet; gl, glume; ra, rachis.

The primer sequences employed for qPCR were based on those targeting the *Btr2* locus present (Supplementary Figure S3), and barley *Actin* (GenBank accession AK362208) was used as the reference sequence. According to a qPCR assay, transcript of both *Btr2-D-1* and *-D-2* was present in spikes sampled at the white anther stage (Figure 5). When RNA *in situ* hybridization was employed using an antisense version of the *Btr2-D-1* sequence as probe, signal was observed below the rachis node and spikelets, and also just above the rachis node (Figure 6A); equivalent signals were not detected when the sense probe was employed (Figure 6B). The same experiment using the *Btr2-D-2* sequence as probe produced the same overall result (Figures 6C,D).

## Discussion

### The Abscission Zone in *Aegilops tauschii* Forms Below the Rachis Node

*Aegilops tauschii* produces barrel type dispersal units by disarticulating below the rachis node. The point of breakage is ensured by the formation of an abscission zone in which an array of lignified cells is formed. A similar structure has been described in the spikes of both *Elymus sibiricus* (Zhao et al., 2017) and *Brachypodium distachyon* (Yu et al., 2020a), whereas the abscission zone cells in the panicles of both wild rice (*Oryza nivara*; Li et al., 2006) and wild sorghum (*Sorghum virgatum*; Lin et al., 2012) are not lignified. In wild *Hordeum* spp., by contrast, which form wedge type units, disarticulation is achieved by the formation of a layer of thin-walled cells in the separation zone (Pourkheirandish et al., 2015). As suggested by (Zhao et al., 2019), the mechanistic basis of rachis brittleness in the genus *Triticum* lies in repressing the synthesis of cell walls. The disarticulation surface in these species is characterized by cells not capped by a cell wall. In *Ae. tauschii*, the separation surface was similarly smooth, but, with the exception of vascular elements, the cells at the surface were capped by an undamaged cell wall. This phenotype resembles that of the separation layer seen in the rachilla of *Elymus sibiricus* (Zhao et al., 2017) and at the base of the pedicel in *Oryza* spp. (Konishi et al., 2006).

### The Ectopic Expression of *Btr2* May Explain the Formation of the Abscission Zone Below the Rachis Node

The co-expression of *Btr1* and *Btr2* is required for disarticulation to occur above the rachis node (Pourkheirandish et al., 2015; Zeng et al., 2020a). The expression of *Btr2* above the rachis node determines where the disarticulation layer forms. *Btr2* is not expressed below the rachis node in species which form wedge type disarticulation units (Pourkheirandish et al., 2015; Zeng et al., 2020a). The genome sequence of *Ae. tauschii* ssp. *strangulata* (Luo et al., 2017) lacks a copy of *Btr1*, and the assumption is that this is similarly the case in *Ae. tauschii* ssp. *tauschii*. The reason why *Ae. tauschii* fails to form a disarticulation layer above the rachis node is presumed to be the absence of an intact copy of *Btr1*, while its formation of a disarticulation layer below the rachis node reflects the operation of a cell-cell separation mechanism, as demonstrated here. The RNA *in situ* hybridization experiment confirmed that there was an abundance of *Btr2* transcript in the abscission zone below the rachis node, which implies that the ectopic expression of *Btr2* is required for the formation of this structure.

The genome of *Ae. longissima* (which does not form barrel type disarticulation units) harbors two copies of *Btr2*, neither of which is transcribed below the rachis node (Zeng et al., 2020a). The implication is that the unusual site of *Btr2* transcription shown by *Ae. tauschii* is a recently acquired trait. An intriguing question is how this product can operate, given that its function is to ensure that disarticulation occurs below the rachis node. A further possibility is the formation of the abscission zone requires not just the product of *Btr2* but also that of a gene(s) such as the ortholog of the rice locus *qSH1* to achieve cell-cell separation in the abscission zone (Konishi et al., 2006; Katkout et al., 2015). Abscission zone is formed above glumes in *Brachypodium*, above the rudimentary glumes and below the empty glumes (sterile lemmas) in rice (Yoshida and Nagato, 2011; Zhou et al., 2012), and below glumes in *Setaria* (Yu et al., 2020a). Though they share distinct abscission zone, *qSH1* is transcribed in each site corresponded to the abscission zone (Yu et al., 2020a). Thus, *qSH1* appears to be a strong candidate to collaborate with *Btr2* to form the abscission zone in *Ae. tauschii*.

## Data Availability Statement

The original contributions presented in the study are included in the article/Supplementary Material, further inquiries can be directed to the corresponding author.

## Author Contributions

XZ and TK planned and designed the research and drafted the manuscript. XZ performed the morphological analysis, the DNA sequencing and the qPCRs, while AT and XZ performed the RNA *in situ* hybridization experiments. HS, SK, and TK monitored the acquisition of data. Each of the authors has read and agreed to the submitted version of the manuscript.

### Conflict of Interest

The authors declare that this research was conducted in the absence of any commercial or financial relationships which could be construed as a potential conflict of interest.

## Footnotes

^1^www.ipk-gatersleben.de/en/genebank/

^2^www.clcbio.com/
