# Supplementary material for: The Ectopic Expression of Btr2 in Aegilops tauschii Switches the Disarticulation Layer From Above to Below the Rachis Node
Source: Front Plant Sci. 2020 Nov 9;11:582622. doi: 10.3389/fpls.2020.582622 (PMC7680762; doi:10.3389/fpls.2020.582622)
Supplement: Supplementary file 1 [file Data_Sheet_1.pdf]

Supplementary Figures

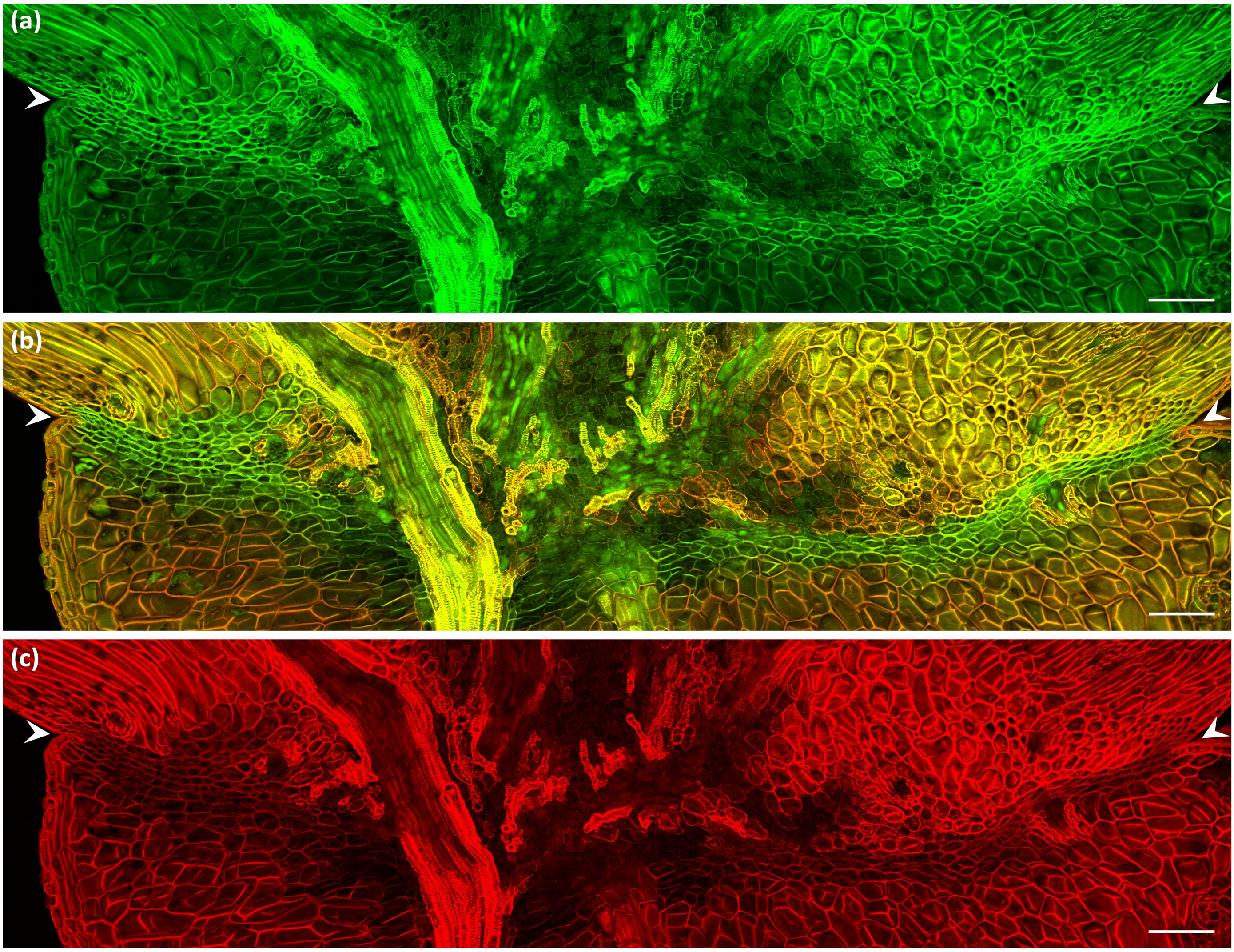

Fig. S1. A close-up view of the abscission zone of *Ae. tauschii* shown in Fig. 3a. The images represent the output measured at (a) 505-530 nm, (c) >600 nm. A merged image is shown in (b). The abscission zone is indicated by white arrows. Scale bar: 100 µm.

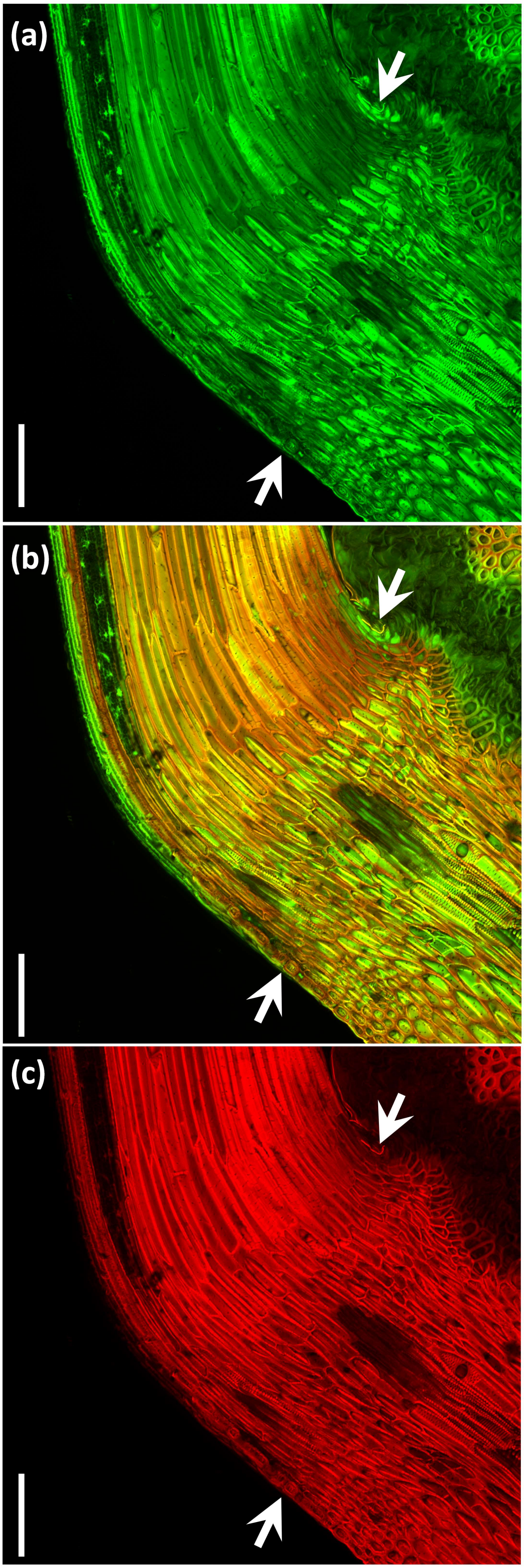

Fig. S2. A close-up view of the disarticulation point of *Ae. longissima* shown in Fig. 3c. The images represent the output measured at (a) 505-530 nm, (c) >600 nm. A merged image is shown in (b). The disarticulation layer is indicated by white arrows. Scale bar: 100 μm.

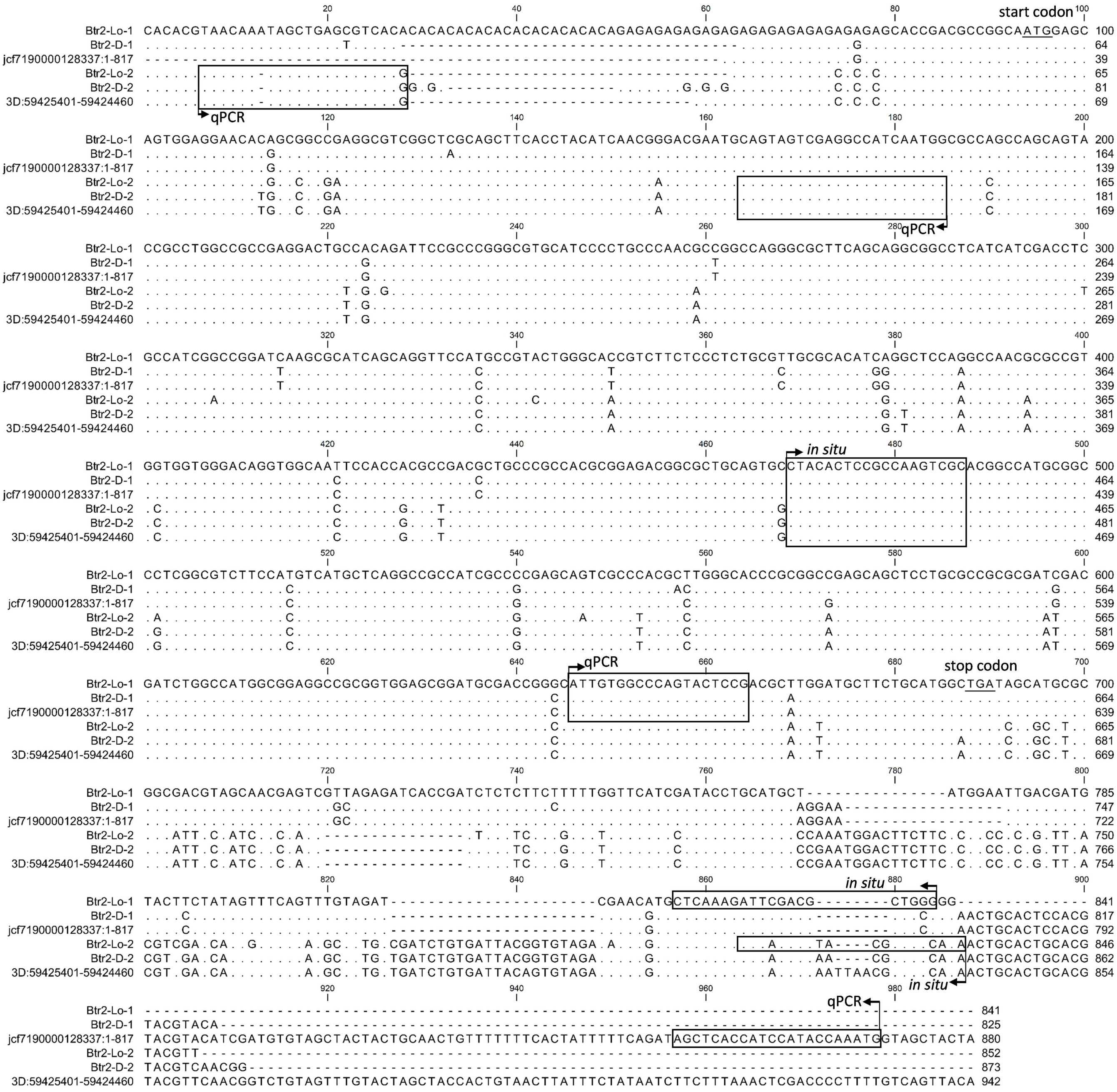

Fig. S3. Alignment of nucleotide sequence between the *Btr2* copies present in *Ae. tauschii* AL8/78 (jcf7190000128337:1-817 and 3D:59425401-59424460) and *Ae. tauschii* AE 956 (*Btr2-D-1* and *-D-2*), and two *Ae. longissima Btr2* copies (*Btr2-Lo-1* and *-Lo-2*). The location of the primers used for qPCRs and the probes used for RNA *in situ* hybridization are boxed.
